# Myocardial remodeling after kidney transplantation: a case report

**DOI:** 10.1186/s12882-018-1185-x

**Published:** 2018-12-20

**Authors:** Marceli Lukaszewski, Kinga Kosiorowska, Dorota Kaminska, Marta Obremska, Oktawia Mazanowska, Magdalena Krajewska

**Affiliations:** 1 Department of Anaesthesiology and Intensive Therapy, Wroclaw Medical University, Borowska 213, 50-556 Wroclaw, Poland; 2 Department of Cardiac Surgery, Wroclaw Medical University, Wroclaw, Poland; 3 Department of Nephrology and Transplantation Medicine, Wroclaw Medical University, Wroclaw, Poland

**Keywords:** SLE, Kidney transplantation, Myocardial remodeling, Levosimendan, Strain

## Abstract

**Background:**

Lupus nephritis (LN) is one of the most common manifestations of systemic lupus erythematosus (SLE) and is often the most serious organ complication and the cause of premature death of such a patient. Most of other organs and systems can be also affected. A typical complication is a cardiovascular involvement leading to the development of heart failure. According to current therapeutic standards, kidney transplantation is the treatment of choice in patients with renal failure in course of LN. On the contrary, a kidney transplantation in a patient with an additional heart disease poses a serious clinical challenge.

**Case presentation:**

We present a case of a 49-year-old woman with renal and heart failure following a long-term SLE prepared for kidney transplantation. During the SLE course, the function of the heart and kidneys gradually deteriorated. The patient required the initiation of renal replacement therapy and was dialyzed until a kidney transplantation for 4 years. In the preparation of the patient for the surgical procedure, due to the extremely low ejection fraction, it was decided to include cardioprotective treatment with Levosimendan. The postoperative period was not straightforward but successful. In the monthly and five-month follow-up, a continuous improvement of heart function with normal renal function was noted.

**Conclusions:**

Kidney transplantation in patients with lupus suffering from heart failure requires the involvement of a team of specialists. Patients with extremely low ejection fraction in the perioperative period should undergo careful hemodynamic supervision in the intensive care unit. Cardioprotective and thus nephroprotective Levosimendan therapy together with optimal fluid and hemodynamic therapy in the peri-transplant period may be a bridge for heart remodeling after kidney transplantation.

## Background

Systemic lupus erythematosus (SLE) is a disease characterized by overactivity of the immune system manifested by the loss of autotolerance with overproduction of autoantibodies leading to multi-organ dysfunction. Kidney involvement (Lupus Nephritis - LN), is often the most serious organ complication and the cause of early death of the patient [1]. Most of other organs and systems can be also affected, sometimes simultaneously co-creating the clinical presentation of the disease. A typical complication is a cardiovascular involvement including valvular heart disease, endocarditis named Libman-Sacks disease, myocarditis, pancarditis, and even heart failure. The inflammatory process may include the heart conduction system and the coronary vascular endothelium promoting the development of early coronary atherosclerosis, leading to myocardial infarctions occurring at a very young age. Heart failure in patients with SLE is complicated by the presence of multiple mechanisms as well as concomitant diseases associated with the underlying disease. An important and common complication is pulmonary hypertension, whether primary due to the direct involvement of the pulmonary vasculature or secondary to chronic thromboembolism or interstitial lung disease. Pulmonary hypertension is a serious obstacle limiting the possibility of treating renal or cardiovascular failure with organ transplantation [2, 3]. Pulmonary hypertension found before renal transplantation is associated with a three-fold higher risk of graft dysfunction [2]. In the past, the increased immunoreactivity in patients with active SLE followed by the high risk of graft rejection excluded them from organ transplantation considerations. However, according to current therapeutic standards, kidney transplantation is the treatment of choice in patients with renal failure in the course of LN [4].

Kidney transplantation in a patient with a baseline cardiac dysfunction is a serious clinical problem. The pathophysiological interaction between the heart and the kidneys, also known as the cardio-renal syndrome (CRS), often occurs in heart failure and is associated with deterioration of renal function, as well as worse prognosis of therapy and quality of life. Considering the high risk of cardiological burdens and, thus, the possible impaired graft perfusion, there is a discussion about whether there are a validity and chance for graft durability and how high the risk of postoperative complications will be [5, 6]. Prospective and retrospective studies showed a significant improvement in cardiac function after kidney transplantation [7, 8] even in cases of pronounced cardiac failure [9–11].

Levosimendan is one of the inodilators. Its inotropically positive action is associated with a decrease in systemic resistance, which is often associated with the need to use vasoconstrictive drugs. Clinical data on the protective effect of Levosimendan on kidney function after various types of surgery are suggestive, but they are based only on a limited number of studies performed on small groups of patients [12, 13].

## Case presentation

**Fig. 1:**
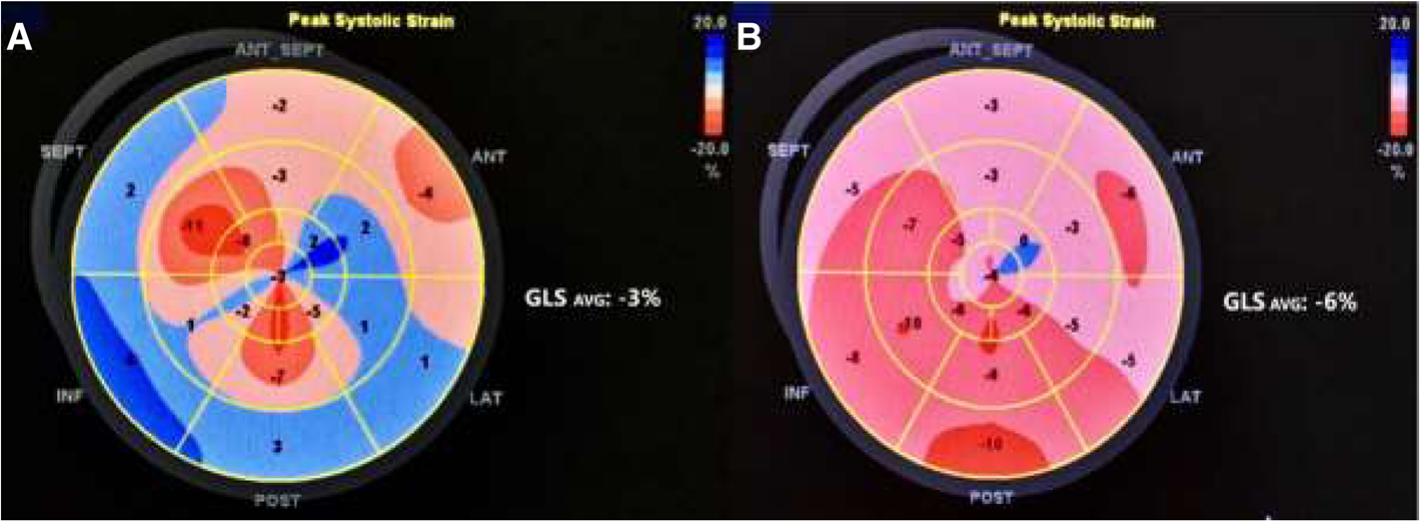
Global and segmental longitudinal strain before (**a**) and after (**b**) kidney transplantation. The bull’s eye plot displays the regional value of peak systolic strain in all 17 left ventricle segments. Their average value is defined as a global longitudinal strain (GLS). Red colours indicate shortening strains and blue indicates lengthening strains

We present a case of a 49-year-old woman with renal and heart failure following a long-term (lasting from 13 years of age) SLE prepared for kidney transplantation. Due to LN (class III, then IV), starting at childhood, she was treated with steroids, together with cyclophosphamide, replaced later by methotrexate and then azathioprine. Hence, the partial remission of nephrotic syndrome was achieved and from 2002 the patient did not receive any immunosuppressive therapy. She was also HBV and HCV positive. SLE involvement of circulatory system presented with early coronary atherosclerosis, ischemic heart disease, and myocardial infarction at the age of 20. In 2007, because of deterioration of kidney function with a serum creatinine concentration of 2.2 mg/dL and proteinuria of 2 g/day, the kidney biopsy was performed. The biopsy showed active and sclerotic focal proliferative lupus nephritis nevertheless immunosuppressive therapy was not introduced for the reason of active replication of HCV. The kidney function was gradually deteriorating over time. Despite cardiac intervention (PCI RCA), the patient developed severe post-infarction and dilated cardiomyopathy and required ICD implantation in primary prevention in 2009. Later, on lupus and secondary cardiomyopathic background, the patient developed severe MV and TV regurgitation. For this reason, the patient underwent mitral and tricuspid valve repair and left ventricle volume reduction surgery complicated by low cardiac output syndrome with a need for intra-aortic balloon pump use (2014). In the postoperative period, the kidney function deteriorated, requiring the initiation of renal replacement therapy. The patient has been on dialysis for 4 years. While being on active waiting list for kidney transplantation presented remission of laboratory indices of lupus (complement splits within normal limits: C3–0,93 g/l, C4–0,4 g/l, ANA negative) and persisting circulatory insufficiency with markedly reduced stair-climbing capacity (to one flight of stairs) with elevated BNP 619 pg/ml (n. 0–100). In transthoracic echocardiography, performed before renal transplantation, the left ventricle and the left atrium were significantly enlarged and the left ventricular systolic function was significantly reduced with LVEF 26% and GLS -3 (Fig. 1a). Due to the implantation of the mitral ring, it was not possible to assess the left ventricular diastolic function. The high tricuspid *regurgitant* flow gradient with widened and poorly respiratory mobile inferior vena cava indicated a high probability of pulmonary hypertension. Furthermore, while preparing the patient for the surgical procedure, it was decided to include cardioprotective therapy with Levosimendan. Due to the time frame associated with the transplantation procedure, the drug infusion was started as soon as possible after cross-match results were known, immediately after the dialysis session. The infusion at a dose of 0.1 μg/kg/min was continued after surgery for a total of 24 h. The patient’s anesthesia for kidney transplantation and perioperative care included the aspect of optimizing transplanted kidney perfusion, avoiding the use of renal toxic drugs and those excreted by properly functioning kidneys, as well as the use of nephroprotective agents. Because of the patient’s cardiological burden, including recurrent episodes of extrasystole proceeding with decompensation of the circulatory system, together with the need of ICD turning off for the transplantation period, the Swan-Ganz catheter for hemodynamic assessment was not used. Anesthesia monitoring was limited the to ECG, central catheter with CVP assessment, direct blood pressure measurement from the cannula inserted into the radial artery, and cardiac ultrasound. In the perioperative period the CVP parameter was used to assess the volatility, and in the postoperative period, a cardiac ultrasound was used along with the assessment of VCI respiratory fill and motility. The therapy was aimed at the standard of fluid therapy called Goal Directed Therapy (GDT) [14, 15]. During general anesthesia, fentanyl, triacrium, propofol, desflurane, antibiotic therapy, and standard immunosuppressive treatment were used as well as 25 g of mannitol infusion was administered as a nephroprotective treatment and 0.9% NaCl as a fluid therapy [15]. In the course of postoperative immunosuppression, she received steroids, tacrolimus with mycophenolate mofetil which was stopped due to persistent leukopenia and cytomegalovirus infection. Furthermore delayed graft function was observed with a need for hemodialysis for almost 6 weeks (mostly due to fluid retention). BNP levels raised to 2996 pg/ml and then slowly decreased. The kidney biopsy performed 2 weeks after transplantation revealed acute rejection (AR II B Banff 2015) with ATN. Finally, the patient was discharged from the hospital on the 67th POD with the serum creatinine concentration of 1.4 mg/dL and BNP level of 1794 pg/ml. One month after kidney transplantation, there was a reduction in left ventricular dimensions, improved systolic function in the EF (increase to 30%) and GLS (decrease to − 6) assessment (Fig. 1b). In addition, there was a decrease in the tricuspid regurgitant flow gradient with normal width and respiratory motility of the IVC, which indicates a low probability of pulmonary hypertension. The improvement of echocardiographic parameters also reflected the simultaneous improvement of exercise capacity in the recipient from NYHA III/IV to NYHA II. In the 5-month observation, further improvement of heart function with a drop of BNP to 1066 pg/ml and normal kidney function were noted.

## Discussion and conclusions

Although in SLE patients kidney transplantations are connected with significantly lower morbidity and mortality than in SLE dialysis patients, the co-morbidities, especially circulatory system involvement remains a significant problem affecting the choice of renal replacement therapy [16]. Unlike bone marrow or kidney, heart or lung transplants are rarely performed in patients with SLE. Case reports of positive posttransplant outcome were published, however, SLE patients are traditionally excluded from transplantation [17]. Lupus-related heart complication with the burden of previously used long-term immunosuppressive treatment makes those patients extremely difficult to manage in the peritransplant period. However, the research confirms that patients suffering from heart failure or coronary artery disease should not be disqualified from kidney transplantation. The number of perioperative cardiac complications is higher in those recipients, but it is within the acceptable range, while in the postoperative period the survival rate of these patients is significantly lower than in patients who have been exclusively dialyzed [6]. Similar survival of the graft is also confirmed in comparable groups of patients with and without left ventricular dysfunction. An important benefit of the procedure is cardiovascular improvement caused by myocardial remodeling with improved systolic heart function [8, 18, 19]. It was shown that amelioration of uremia after kidney transplantation improved cardiac function in patients with dilated cardiomyopathy and long dialysis period [7]. In patients with left ventricular dysfunction, the post-transplantation mean LVEF was reported to increase significantly from 41 to 50% with the improvement in other parameters, including diastolic function, LV end-diastolic dimension, LV mass, and right ventricular systolic pressure [20]. Wali et al. in the prospective study showed the remodeling with the further improvement of cardiac systolic function in the period of 6 and 12 months after surgery [10]. The similar observation was presented by Melchor et al., wherein the group with initially impaired left ventricular function the improvement of cardiac index and contractility after the first post-transplant month followed by further improvement in left ventricular ejection fraction after 12 months was reported [11]. Patients with extremely low ejection fraction are not often considered for kidney transplantation because of the high risk of peri-operative complications. In the presented case we decided to include Levosimendan therapy during kidney transplantation procedure. Levosimendan is one of the inodilators. Its inotropically positive action is associated with a decrease in systemic resistance, which is often related to the need to use vasoconstrictive drugs. The mechanism of action is mainly by increasing the sensitivity of myocytes to calcium by selectively binding calcium ions to troponin C. This increase in sensitivity and improvement in the contractility of cardiac muscle cells is not due to increased intracellular calcium or oxygen consumption, unlike other inotropically positive drugs. Another mechanism of action of the drug is the opening of ATP-dependent potassium channels in smooth muscle cells, which results in the described reduction of flow resistance through the veins and arteries. Hence, the opening of ATP-dependent potassium channels in cardiomyocytes is expected to have both cardioprotective and nephroprotective effects. Twenty-four hour infusion causes the described hemodynamic effect to persist for many days after its completion, which is the result of the slow elimination of its active metabolites [21, 22].

The pathological interaction between the heart and the kidneys, also known as the cardio-renal syndrome (CRS), often occurs in heart failure and is associated with deterioration of renal function, as well as worse prognosis of therapy and quality of life. Clinical data on the protective effect of Levosimendan are suggestive, but they are based only on a limited number of studies performed on small groups of patients. Improvement occurs through the described vasodilation, increased renal artery diameter, increased renal blood flow and simultaneous improvement of cardiac output. An equally important element of nephroprotection is the action of Levosimendan which improves the function of the right ventricle with a decrease in pulmonary hypertension and venous stasis within the kidneys [1, 13, 23]. Randomized studies in patients with left ventricular dysfunction (REVIVE I and II, CHEETAH, LICORNE) and the recent CTS-LEVO study, failed to clearly confirm the effectiveness of Levosimendan therapy. In CHEETAH and LICORN studies, statistically significant improvement in patient survival and organ function improvement was not confirmed, only in the LEVO-CTS study, in one of the endpoint, it was confirmed that prophylactic Levosimendan infusion initiated immediately before surgery improves left ventricular function after cardiac surgery [1]. However, the abovementioned studies were carried out on a group of cardiac surgery patients and were burdened with a number of limitations, such as the too low dose of the drug, no loading dose, and delayed start of the infusion. In the aspect of numerous observations made earlier and in the most recent studies, it is believed that Levosimendan therapy should be used in selected groups of patients, and further research on the use of this drug should be continued [1]. In addition, in the preliminary animal studies described in the literature, a reduction in oxidative stress is observed in the ischemia-reperfusion syndrome through the use of Levosimendan, which is related to its effects on the nitric oxide metabolism [1, 24–26]. In the post-transplant period, the inclusion of immunosuppressive drugs may be associated with an increase in BMI and an increase in blood pressure, and thus an afterload increase and cardiac function deterioration [10]. For this reason, Levosimendan therapy seems to be particularly beneficial due to the vasodilatation effect and afterload reduction.

To conclude, kidney transplantation in patients with lupus suffering from heart failure each time requires an individual approach in the form of teamwork, which includes specialists in the field of nephrology and transplantology, cardiology and intensive care, as well as the extension of therapeutic concepts with the latest possibilities in the field of applied therapies. Patients with extremely low EF < 30% in the perioperative period should undergo careful hemodynamic supervision in the intensive care unit. Cardioprotective and thus nephroprotective Levosimendan therapy together with optimal fluid (GDT) and hemodynamic therapy in the peri-transplant period may be a bridge for heart remodeling after kidney transplantation.

## Abbreviations

ANA: antinuclear antibody
ATN: acute tubular necrosis
ATP: adenosine triphosphate
BMI: body mass index
BNP: brain natriuretic peptide
CRS: cardio-renal syndrome
CVP: central venous pressure
ECG: electrocardiogram
FSGS: focal segmental glomerulosclerosis
GDT: goal directed therapy
GLS: global longitudinal strain
HBV: hepatitis B virus
HCV: hepatitis C virus
ICD: implantable cardioverter defibrillator
LN: lupus nephritis
LV: left ventricle
LVEF: left ventricle ejection fraction
MV: mitral valve
NYHA: New York Heart Association
PCI: percutaneous coronary intervention
POD: postoperative day
RCA: right coronary artery
SLE: systemic lupus erythematousus
TV: tricuspid valve
VCI: vena cava inferior
